# Transcriptomic Modulation Reveals the Specific Cellular Response in Chinese Sea Bass (*Lateolabrax maculatus*) Gills under Salinity Change and Alkalinity Stress

**DOI:** 10.3390/ijms24065877

**Published:** 2023-03-20

**Authors:** Qing Zhu, Moli Li, Wei Lu, Yapeng Wang, Xujian Li, Jie Cheng

**Affiliations:** 1 Key Laboratory of Marine Genetics and Breeding (Ocean University of China), Ministry of Education, 5 Yushan Road, Qingdao 266003, China; 2 Key Laboratory of Tropical Aquatic Germplasm of Hainan Province, Sanya Oceanographic Institution, Ocean University of China, Sanya 572024, China; 3 Laboratory for Marine Fisheries Science and Food Production Processes, National Laboratory for Marine Science and Technology (Qingdao), 1 Wenhai Road, Qingdao 266237, China

**Keywords:** saline-alkaline adaptation, oxidative stress, osmotic stress, co-expression network, *Lateolabrax maculatus*

## Abstract

Salinity and alkalinity are among the important factors affecting the distribution, survival, growth and physiology of aquatic animals. Chinese sea bass (*Lateolabrax maculatus*) is an important aquaculture fish species in China that can widely adapt to diverse salinities from freshwater (FW) to seawater (SW) but moderately adapt to highly alkaline water (AW). In this study, juvenile *L. maculatus* were exposed to salinity change (SW to FW) and alkalinity stress (FW to AW). Coordinated transcriptomic responses in *L. maculatus* gills were investigated and based on the weighted gene co-expression network analysis (WGCNA), 8 and 11 stress-responsive modules (SRMs) were identified for salinity change and alkalinity stress, respectively, which revealed a cascade of cellular responses to oxidative and osmotic stress in *L. maculatus* gills. Specifically, four upregulated SRMs were enriched with induced differentially expressed genes (DEGs) for alkalinity stress, mainly corresponding to the functions of “extracellular matrix” and “anatomical structure”, indicating a strong cellular response to alkaline water. Both “antioxidative activity” and “immune response” functions were enriched in the downregulated alkaline SRMs, which comprised inhibited alkaline specific DEGs, revealing the severely disrupted immune and antioxidative functions under alkalinity stress. These alkaline-specific responses were not revealed in the salinity change groups with only moderately inhibited osmoregulation and induced antioxidative response in *L. maculatus* gills. Therefore, the results revealed the diverse and correlated regulation of the cellular process and stress response in saline-alkaline water, which may have arisen through the functional divergence and adaptive recruitment of the co-expression genes and will provide vital insights for the development of *L. maculatus* cultivation in alkaline water.

## 1. Introduction

Salinity and alkalinity are important aquatic environmental factors that can directly affect the cellular physiology and immune response of aquatic species [1,2,3]. Teleost species that can adapt to altered salinity are highly species-specific, and they could be classified into euryhaline (tolerate high fluctuation of salinity) or stenohaline (tolerate low fluctuation of salinity) [4], whereas high alkalinity is harmful to most fish species. For instance, a salinity change can increase lysozyme activity, mucosal production and antioxidative and immune defense in teleosts, while high alkalinity could lead to synergistic toxicity of total alkalinity, pH and the resulting changes in the metabolism of ammonia nitrogen, which could cause the degradation of biomolecules with increased mortality rates [1,5,6,7]. Moreover, under salinity change and alkalinity stress, energy metabolism increases to maintain the osmotic balance in aquatic species [8]. However, excessive energy metabolism may lead to increased oxidation levels, resulting in the accumulation of reactive oxygen species (ROS), which may trigger oxidative stress damage [4,9], impair cell function and allow free radicals (e.g., ROS) to cause lipid peroxidation [10], whereas the antioxidative enzymes (SOD, CAT and GPX) can cope with the increased ROS [11,12]. Furthermore, salinity change and alkalinity stress can also affect ion regulation in aquatic organisms, and the gills play an essential role in maintaining the ion-base balance with specific ion transporters expressed on the apical surface of mitochondria-rich cells [3,13]. Thus, salinity change and alkalinity stress can disrupt ion transport and transport-related enzymes, causing alternations in the gill filaments and lamellae structure in teleost species [14]. Therefore, tissue investigation in fishes represents a valuable tool to assess their health status, which could aid in the study of the relationship between tissue properties and habitat, as well as the species’ ability to adapt to the environment [15,16,17,18,19,20].

Chinese sea bass (*Lateolabrax maculatus*) is an economically important marine fish widely distributed in the coastal and estuarine areas of China, Japan and the Korean peninsula with high nutritional value [21,22]. *L. maculatus* shows an excellent ability to adapt to a broad variety of salinity environments ranging from freshwater to seawater [23,24], therefore, its aquaculture is viable in both freshwater ponds and seawater cages in China. Additionally, *L. maculatus* has been proven to be able to survive in highly alkaline (10 mmol/L) water for a long period of time [25,26], and the aquaculture of *L. maculatus* in alkaline water has also been developed recently. Therefore, with saline-alkaline water aquaculture becoming a promising way to accommodate the growing need for aquaculture, the investigation of salinity and alkalinity stress tolerance in aquatic species is vitally important for the screening of alkaline-tolerant species, and it is essential for the fishery industry to investigate the physiological changes in fishes adapting to saline-alkaline waters and to understand their adaptation in the aquatic ecosystem [1,6].

Recently, although numerous studies have strongly implicated the essential role of cellular physiology as well as antioxidative and immune enzymes in saline-alkaline adaptation [27,28,29], many of these studies are only focused on responses for a limited number of genes rather than the full complement of gene sets that comprise the complex network in saline-alkaline adaptation [25,30,31,32]. Weighted gene co-expression network analysis (WGCNA) is a promising method that can provide an integrated view of the gene interaction cascade even through date cross-species and cell types [33,34]. WGCNA can detect clusters of highly correlated genes and reveal not only the implication of previously studied genes but also pathways unconsidered so far [35,36]. Therefore, the combination of gene expression and network analysis will provide a more comprehensive indication of how teleost species respond to dynamic environmental challenges in terms of cellular signaling pathways [23,24,26].

To explore the different molecular responses of *L. maculatus* under salinity change and alkalinity stress, in this study, a transcriptome and modulated co-expression gene network analysis were performed with the gill tissues of *L. maculatus*. The effects of salinity and alkalinity on the aspects of osmoregulation, antioxidative and immune responses, as well as the associated biochemical and molecular events in *L. maculatus* are investigated. The results will provide comprehensive insights for further understanding the environmental adaptation of *L. maculatus* to salinity change and alkalinity challenge and will help to assess the potentiality of *L. maculatus* in saline-alkaline aquaculture.

## 2. Results and Discussion

### 2.1. Histopathological Alteration of L. maculatus Gills under Salinity Change and Alkalinity Stress

**Figure 1:**
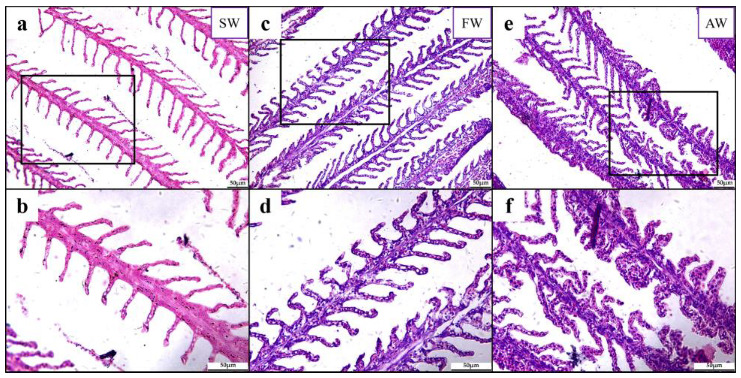
Effects of salinity change and alkalinity stress on *L. maculatus* gills with H&E stain. (**a**,**b**) are the seawater (SW) group; (**c**,**d**) are the freshwater (FW) group; (**e**,**f**) are the alkaline water (AW) group. The pictures are shown as 20× view (upper panels) and enlarged from the selected frame regions as 40× view (lower panels).

In teleost fishes, the gill filaments and lamellae are typically long and thin to maximize surface area and minimize diffusion distances between water and blood [37]. There were intact gill filaments and lamellae structures without damage observed in the *L. maculatus* gills of the seawater (SW) group (Figure 1a,b), while the *L. maculatus* from the freshwater (FW) group showed slight swelling of gill lamellae and epithelial cells (Figure 1c,d). Moreover, deformation, strong swelling of the gill filaments and lamellae and obvious fusion of epithelial cells were found in the *L. maculatus* of the alkaline water (AW) group (Figure 1e,f). Therefore, compared to seawater, both freshwater and alkaline water could induce varying degrees of *L. maculatus* gill structure changes, especially in the alkaline water group.

### 2.2. Transcriptomic Response of L. maculatus Gills to Salinity Change and Alkalinity Stress

To characterize the transcriptomic response of *L. maculatus* gills to salinity change and alkalinity stress, 9 and 12 *L. maculatus* gill RNA-seq libraries were analyzed, respectively. Firstly, compared to the seawater (SW) group, a total of 61 (20 up and 41 down) and 136 (58 up and 78 down) differentially expressed genes (DEGs, |log_2_FC| ≥ 1.5 and *FDR* < 0.05) were identified in the hypotonic salinity changes of the brackish water (BW) and freshwater (FW) groups, respectively (Figure S1). Specifically, the upregulated DEGs were enriched in GO terms including “transmembrane transporter activity”, “small molecule metabolic process”, “response to stimulus”, “oxidoreductase activity” and “response to oxidative stress” et al. (Figure S1 and Table S1), whereas the downregulated DEGs mainly enriched GO terms such as “transmembrane transporter activity”, “response to stimulus”, “response to osmotic stress”, “inflammatory response” and “regulation of apoptotic signaling pathway” et al. (Figure S1 and Table S1). In addition, compared to the FW group (0 h), a total of 89 (71 up and 18 down), 159 (88 up and 71 down) and 763 (567 up and 196 down) DEGs (|log_2_FC| ≥ 1.5 and *FDR* < 0.05) were identified for the 12 h, 24 h and 72 h of hypertonic alkaline water (AW) challenges, respectively (Figure S2). Most DEGs were from the 72 h AW group, and more specifically, the upregulated DEGs were enriched in GO terms including “extracellular matrix”, “anatomical structure”, “cell adhesion”, “intermediate filament” and “neurogenesis” et al. (Figure S2 and Table S1), whereas the downregulated DEGs mainly enriched GO terms such as “actin/myo- filament”, “response to stress”, “cell cycle”, “myosin complex” and “response to bacterium” et al. (Figure S2 and Table S1). These regulated DEGs indicated that, even well adapted to the hypotonic salinity change in FW, there were regulated antioxidative and immune functions in *L. maculatus* gills, whereas with the hypertonic alkalinity challenge, more significant cellular process alterations and stress effects were obtained, which could be observed from the damaged gill filaments and lamellae structures (Figure 1e,f).

**Figure 2:**
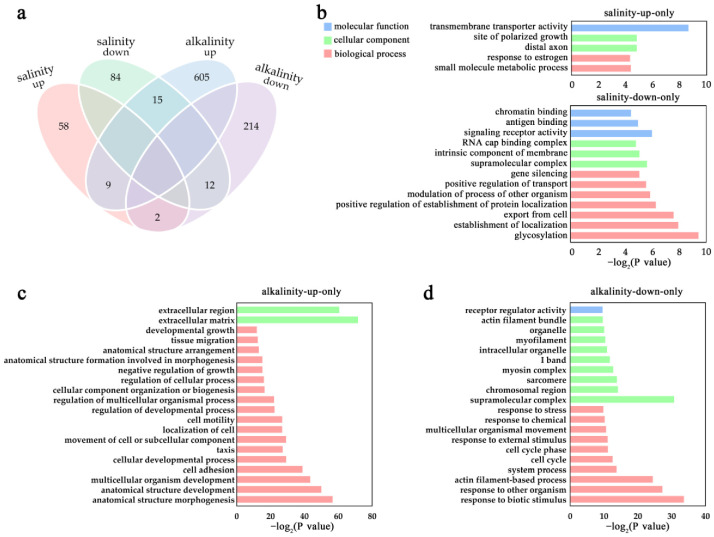
Differential gene expression in *L. maculatus* gills with the combined salinity and alkalinity challenges. (**a**) DEGs shown as the Venn diagram between the salinity and alkalinity challenges; (**b**) Functional GO enrichment of the salinity-specific DEGs at level 3; (**c**,**d**) Functional GO enrichment of the alkalinity-specific DEGs at level 3.

To differentiate the specific transcriptomic response of *L. maculatus* gills under salinity change (hypotonicity) and alkalinity stress (hypertonicity), the DEGs from the two conditions were further compared. Interestingly, a total of 58, 84, 605 and 214 DEGs (|log_2_FC| ≥ 1.5 and *FDR* < 0.05) were identified specifically for groups of FW-up, FW-down, AW-up and AW-down, respectively (Figure 2a). In detail, the FW-up specific DEGs were enriched in GO terms of “transmembrane transporter activity”, “lipoprotein oxidation”, “ion transport” and “cilium or flagellum-dependent cell motility” et al. (Figure 2b and Table S2), whereas the FW-down specific DEGs mainly enriched GO terms such as “response to osmotic stress”, “transport” and “transmembrane signaling receptor activity” et al. (Figure 2b and Table S2). In addition, the AW-up specific DEGs enriched GO terms of “extracellular matrix/region”, “anatomical structure”, “cell adhesion”, “neurogenesis” and “cell development/differentiation” et al. (Figure 2c and Table S2), while the AW-down specific DEGs mainly enriched GO terms including “responses to stimulus”, “actin filament”, “myosin complex”, “cell cycle” and “muscle tissue/organ development/morphogenesis” et al. (Figure 2d and Table S2). There were only a few overlapped DEGs shared between groups (Figure 2a and Figure S3), and the abundant saline/alkaline specific DEGs may indicate the particular gene sets participating in the diverse hypo- or hyper-tonic osmoregulation and cellular processes in *L. maculatus* gills.

### 2.3. Co-Expression Network of L. maculatus Genes under Salinity Change and Alkalinity Stress

**Figure 3:**
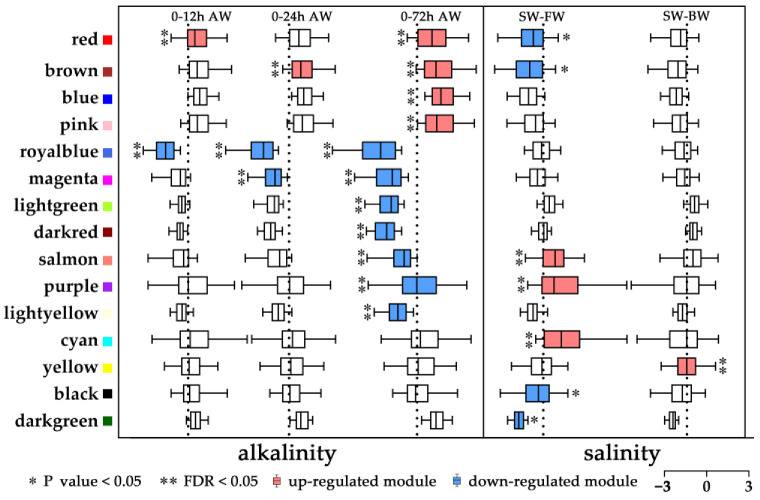
WGCNA in *L. maculatus* gills with salinity change and alkalinity stress. Stress-responsive modules (SRMs), defined with different colours, were identified by enrichment analysis of DEGs with salinity change and alkalinity stress. Red and blue boxes indicate up- and down-regulated modules, respectively.

To further understand the modulated gene regulation and interaction in gills of *L. maculatus* under salinity change and alkalinity stress, 16,864 expressed genes from the combined 21 transcriptome samples (salinity and alkalinity) were used to perform the WGCNA [35], and they were assigned into 23 modules with the size ranging from 71 to 2938 genes (Figure 3, Figure S4 and Table S3). The stress-responsive modules (SRMs) were identified based on the overrepresentation of DEGs using a hypergeometric test. As a result, 15 SRMs were identified (*FDR* or *p* < 0.05), including 8 and 11 modules enriching saline- and alkaline-response DEGs, respectively (Figure 3 and Table S4). Among the 8 saline SRMs, 4 modules (salmon, purple, cyan and yellow) enriched upregulated DEGs (*FDR* < 0.05), 4 modules (red, brown, black and dark green) enriched downregulated DEGs (*p* < 0.05), whereas for the 11 alkaline SRMs, 4 modules (red, brown, blue and pink) enriched upregulated DEGs and 7 modules (royal blue, magenta, light green, dark red, salmon, purple and light yellow) enriched downregulated DEGs (*FDR* < 0.05) (Figure 3 and Table S4). Interestingly, among the 15 SRMs, 4 modules represented overlapping but opposite responsive patterns between saline and alkaline changes, with the red and brown modules upregulated in AW but downregulated in FW, whereas the salmon and purple modules upregulated in FW but downregulated in AW (Figure 3), which may indicate the diversified regulation patterns under the hypo- or hyper-tonic stresses.

### 2.4. Coordinated Regulation of Saline and Alkaline Specific L. maculatus DEGs

**Figure 4:**
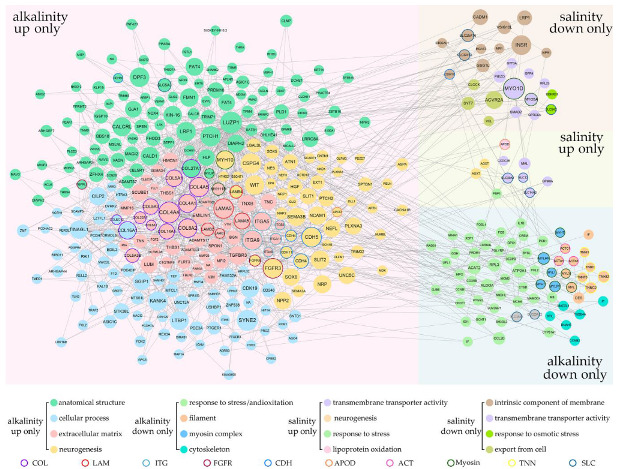
The gene network of DEGs from the hypotonic and hypertonic responsive modules in *L. maculatus* gills. The circle size represents the intramodular connectivity (Kwithin), the circle color represents the annotated GO terms, and the circle frame color represents the different up- and down-regulated responsive DEGs.

A large number of DEGs were specifically regulated under salinity or alkalinity changes (Figure 2a), which resulted in diverse cellular responses in *L. maculatus* gills. These DEGs from enriched GO terms (Table S2) were interlinked in the networks (Figure 4). Therefore, the SRMs and DEGs as key nodes from known and novel pathways were investigated and discussed to illustrate the core gene interactions in hypo- or hyper-tonic specific responses of *L. maculatus* gills. Firstly, several saline-specific DEGs interlinked in the networks were membrane and (ion)-transporter activity-related genes (Figure 2b), such as *solute carrier* transporters (SLC6A, SLC9A, SLC12, SLC14A, SLC20A, SLC25A) being either up- or down-regulated under the FW acclimation (Figure 4 and Figure S5a), which indicated the diverse functions of SLCs in transmembrane transport [38]. Meanwhile, *SLC9A2* and *bradykinin receptor* (BDKRB1) from the “response to osmotic stress” function were both inhibited in the FW group, whereas *alanine-glyoxylate aminotransferase* (AGXT), the peroxisomal aminotransferase that catalyzes the transamination of glyoxylate to glycine and contributes to the glyoxylate detoxification [39] and *apolipoprotein D* (APOD), a transporter involved in lipid trafficking, inflammation and antioxidative response [40], were both upregulated in the FW group (Figure 4). These results suggested that, under FW acclimation, a certain extent of inhibited osmoregulation and induced antioxidative response in *L. maculatus* gills were observed, which was also reported in other studies from fish species like European seabass (*Dicentrarchus labrax*) [4,27,28].

There were more alkaline-specific DEGs in the networks than saline-specific DEGs. For the AW-up specific DEGs, a few “extracellular matrix” and “cell adhesion” related GO terms were enriched (Figure 2c) with *collagen* (COL), *laminin* (LAM) and *integrin* (ITG) genes induced and interlinked in the network (Figure 4 and Figure S5b), indicating activated cellular response under hypertonic alkaline stress. Collagen is a stratified epithelial basement membrane protein that may contribute to epithelial basement membrane organization and adherence by interacting with extracellular matrix (ECM) proteins [41], laminin could mediate the attachment, migration and organization of cells into tissues by interacting with other ECM components [42], while integrin is a large family of ECM receptors as key mediators of cell-matrix and cell-cell adhesion [43]. The tight interaction of these upregulated *collagen*s (COL4, COL5, COL8, COL14, COL16, COL22, COL27), *laminin*s (LAMA5, LAMB2, LAMB4, LAMC3) and *integrin*s (ITGA5, ITGA8, ITGA9, ITGA11, ITGB3) suggested their vital functions in the ECM structure maintain and cell-matrix adhesion under the hypertonic alkaline stress in *L. maculatus* gill filament and lamellae (Figure 4 and Figure S5b). Interestingly, there were “neurogenesis” related GO terms enriched with the AW-up specific DEGs, as *cadherin* (CDH4, CDH5, CDH11) and *fibroblast growth factor receptor* (FGFR3, FGFR4) genes interlinked in the network (Figure 4 and Figure S5b). Cadherins are calcium-dependent cell adhesion proteins and may contribute to the organization of intercellular junctions [44], while FGFR is a tyrosine-protein kinase that acts as a cell-surface receptor for fibroblast growth factors and plays a role in the regulation of cell proliferation, differentiation, apoptosis and migration [45]. These induced and tightly interconnected “extracellular matrix”, “cell adhesion” and “neurogenesis” DEGs contributed to the cellular homeostasis of gill filaments and laminae under the severe hypertonic alkaline stress but not in hypotonic FW changes.

**Figure 5:**
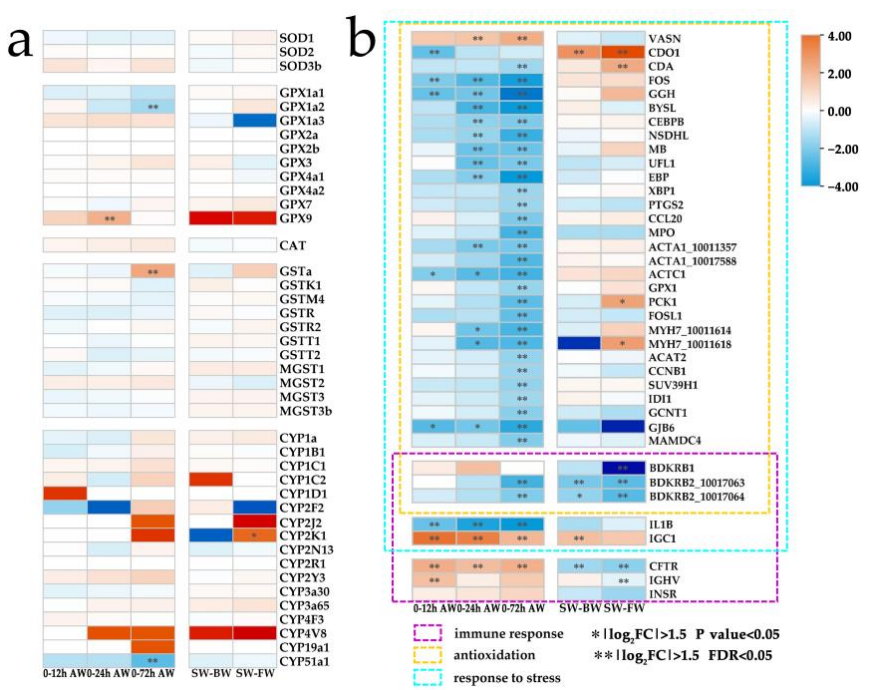
Differential expression of antioxidative genes in *L. maculatus* gills under salinity change and alkalinity stress. (**a**) common antioxidative genes and (**b**) non-conventional antioxidative genes with their GO term clustering.

For the AW-down-specific DEGs, a large number of genes were enriched in the functions of response to diverse stimuli and stresses, among which many DEGs were related to immune and antioxidative responses (Figure 2d and Table S2). Many studies revealed that both hypo- and hyper-tonic salinity may induce oxidative stress mainly due to the low level of redox regulatory enzymes [4]. Normally, the major front-line antioxidative enzymes, such as superoxide dismutase (SOD), catalase (CAT) and glutathione peroxidase (GPX) could neutralize superoxide radicals to H_2_O_2_ and then to water [4]. Glutathione-S transferase (GST) and cytochrome 450 (CYP) are also considered to be redox-helping regulatory enzymes [4,46]. Here, the *SOD*, *GPx*, *CAT*, *GST* and *CYP* genes were systematically identified in the *L. maculatus* genome (Table S5), and a few of these antioxidative enzymes were significantly regulated (Figure 5a), with *cyp2k1* upregulated in the FW group, *gpx9*, *gsta*-induced and *gpx1a2*, *cyp51a1* inhibited in the AW group (Figure 5a). For instance, glutathione peroxidase 1 (GPX1) plays a crucial role as a glutathione peroxidase [47], and the cytochrome P450 monooxygenase (Cyp51A1) is involved in sterol biosynthesis [48]. Moreover, the information obtained here also revealed that other non-conventional genes not considered so far may participate in the antioxidative process in *L. maculatus* gills under alkalinity stress. Several downregulated DEGs were interlinked in the network (Figure 4) with enriched functions of the “oxidation-reduction process” (Figure 5b). For example, *myeloperoxidase* (MPO) is part of the host defense system of polymorphonuclear leukocytes, which potently inhibits oxidation of low-density lipoprotein particles and limits vascular damage [49]; *Acetyl-CoA acetyltransferase* (ACAT2) is involved in fatty acid beta-oxidation [50]; and *cytochrome c oxidase subunit 5B* (COX5B), as the component of the cytochrome c oxidase, drives oxidative phosphorylation [51], which could function in regulating ROS production and immunity.

In addition, immune functions were also enriched in the AW-down DEGs. For example, *PR domain zinc finger protein 1* (PRDM1) mediates a transcriptional program in various innate and adaptive immune tissue types, and therefore may provide immediate immunological protection against reactivating infections [52]; *Interleukin-1 beta* (IL1B) induces T-cell activation and cytokine production, B-cell activation and antibody production, as well as fibroblast proliferation and collagen production [53]. Interestingly, several *bradykinin receptor* genes (BDKRB1, BDKRB2), which could bind the bradykinin and are essential to the regulation of blood pressure, inflammation, coagulation and pain control [54], were downregulated in both the FW and AW groups and were enriched in functions of “immune response”, “oxidative activity”, “response to osmotic stress”, and “(ion)-transporter activity” (Figure 5b and Table S1). This may suggest the essential role of BDKRBs for the first time as factors in osmotic-related immune and antioxidative responses under both hypo- and hyper-tonic stresses and warrant further investigation of their functions in alkalinity adaptation. Therefore, the inhibition of interlinked immune and antioxidative functional DEGs (Figure 4 and Figure 5) suggested that the immune and antioxidative system in *L. maculatus* gills may be disrupted under strong alkalinity stress.

Moreover, “cell cycle” related functions were also inhibited in the AW-down DEGs. For instance, *G2/mitotic-specific cyclin-B* (CCNB1, CCNB2) is essential for the control of the cell cycle at the G2/M (mitosis) transition [55], *DNA replication licensing factor* (MCM2, MCM4) is the replicative helicase essential for ‘once per cell cycle’ DNA replication initiation and elongation in eukaryotic cells [56]. In addition, cellular structure-related functions such as “actin filament”, “myosin complex” and “cytoskeleton” were enriched including *myosin* (MYL1, MYL3, MYH7, MYLPF), *actin* (ACTA1, ACTC1) and *troponin* (TNNC2, TNNT3, TNNI1) genes inhibited and correlated in the network (Figure 4 and Figure S5c), indicating the inhibition of intracellular movements and membrane trafficking in gill filament due to alkalinity stress [57]. Therefore, strongly activated extracellular matrix and inhibited antioxidative and immune functions in the AW group but not in the FW group revealed the more severe effects of AW than FW for *L. maculatus*, which may warrant further investigation of their specific functions in alkalinity adaptations.

### 2.5. Modulation of L. maculatus SRMs with the Salinity and Alkalinity Overlapped Response

**Figure 6:**
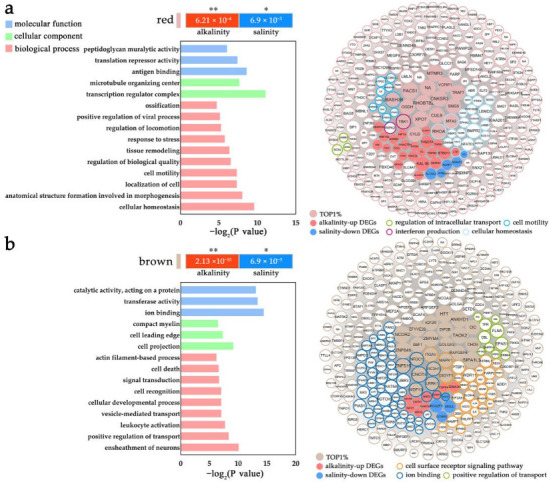
The GO enrichment and gene network of overlapped alkaline-up and saline-down SRMs in *L. maculatus* gills, as (**a**) the red and (**b**) the brown modules with top 300 genes ranking with their connectivity. The circle size represents the intramodular connectivity (Kwithin) and the circle frame color represents the annotated GO terms. * indicate *p* < 0.05, ** indicate *p* < 0.001.

Other than the DEGs coordinately regulated in the networks, the SRMs mainly clustered non-DEGs with enriched vital functions. For example, four SRMs represented overlapped but opposite responsive patterns between saline and alkaline challenges, with the red and brown modules upregulated in AW but downregulated in FW, whereas the salmon and purple modules were upregulated in FW but downregulated in AW (Figure 3 and Table S4). To further understand the functional interaction of genes in specific modules, the 15 SRMs were annotated with GO (Table S6), and the AW and FW overlapping SRMs were investigated. For the AW-up and FW-down modules, the red module represented enriched functions including “interferon production”, “cellular homeostasis”, “anatomical structure”, “intracellular transport” and “cell motility” et al. (Figure 6a and Table S6), while the brown module enriched functions such as “ion/cation binding”, “regulation of transport”, “actin filament” and “cell surface receptor signaling pathway” et al. (Figure 6b and Table S6), both indicating the dynamic activated gene regulation in *L. maculatus* gills under alkalinity stress which was inhibited in freshwater change. For example, in the red module, *cystic fibrosis transmembrane conductance regulator* (CFTR), as an epithelial ion channel that plays an important role in the regulation of epithelial ion/water transport and fluid homeostasis [58], is the significant upregulated DEG in AW but downregulated in FW (Figure S3). The varied CFTR expression was in agreement with that in European seabass (*D. labrax*) under diverse salinity changes [27,28], indicating its specific function in ion transport for osmoregulatory balance under hypo- and hyper-tonic stimuli.

**Figure 7:**
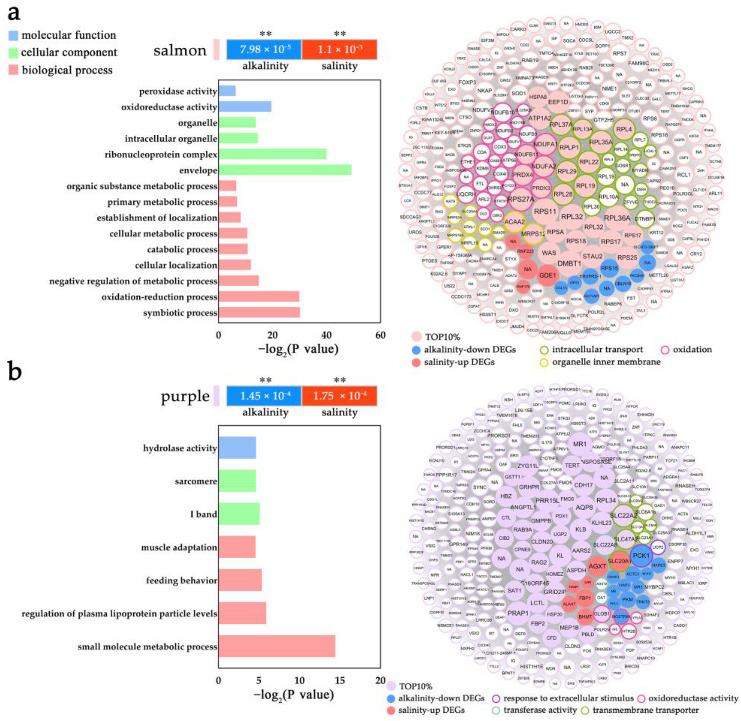
The GO enrichment and gene network of overlapped alkaline-down and saline-up SRMs in *L. maculatus* gills, as (**a**) the salmon and (**b**) the purple modules with top 300 genes ranking with their connectivity. The circle size represents the intramodular connectivity (Kwithin) and the circle frame color represents the annotated GO terms. ** indicate *p* < 0.001.

For the AW-down and FW-up modules, the salmon module represented enriched functions including “ribosomal”, “oxidation”, “organelle inner membrane” and “intracellular transport” et al. (Figure 7a and Table S6), while the purple module mainly enriched functions such as “response to extracellular stimulus”, “transmembrane transporter”, “oxidoreductase activity” and “regulation of NAD(P)H oxidase activity” et al. (Figure 7b and Table S6), both indicating the gene inhibition in *L. maculatus* gills with alkalinity stress but induction with freshwater change. For example, in the salmon module, several *ribosomal protein*s (RPLP1, RPL4, PRL13, RPL19, RPL22, RPL28, RPL29, RPL35A, RPL37A) represented the top hub genes from the “intracellular transport” and “ribosome” functions [59], while *NADH dehydrogenase ubiquinone* (NDUFA1, NDUFA2, NDUFB11) were the key hub genes from the “oxidoreductase activity” function (Figure 7a and Table S6) [60]. Oxidative stress plays essential roles through low or high O_2_ in hyper- or hypo-tonic conditions, respectively [4,61]. Activation of an alternate oxidase system to the low O_2_ level at high salinity (hypertonic) could be the energy-saving and precautionary process in aquatic animals that reduces the enzymes to produce ROS [4,62], while under hypotonic conditions, high O_2_ availability and consumption can follow high respiration events and lead to a high risk of ROS production. The interaction of inhibited antioxidation function further supported the DEG results that the strong alkalinity stress may reduce antioxidative enzyme activities, decrease the ability to scavenge ROS and cause oxidative damage to cells, which functions well in freshwater acclimation (Figure 4).

In addition, candidate AW (two up and five down) and FW (two up and two down) specific SRMs were investigated (Table S6). For instance of the AW-specific SRMs, the upregulated blue module represented enriched functions including “extracellular structure”, “cellular process”, “neurogenesis”, “anatomical structure” and “cell adhesion” et al. (Figure S6a and Table S6), the downregulated royal blue module represented functions of “response to stresses”, “regulation of immune system process”, “leukocyte differentiation” and “interleukin production” et al. (Figure S6b and Table S6), and the downregulated light yellow module represented functions of “cell cycle”, “cellular response to stress” and “regulation of defense response” et al. (Figure S6c and Table S6). These functions agreed with the DEG results that there were activated extracellular matrix and anatomical structures and inhibited antioxidative and immune functions under alkalinity stress. In addition, for the FW-specific SRMs, the upregulated cyan module represented enriched functions including “negative regulation of transport”, “ion channel regulator activity”, “oxidoreductase activity” and “negative regulation of ion transmembrane transport” et al. (Figure S7a and Table S6), the downregulated black module represented functions of “oxidoreductase activity”, “fatty-acyl-CoA metabolic process”, “cellular lipid metabolic process” and “transposition” et al. (Figure S7b and Table S6), and the downregulated dark green module represented functions of “actin filament-based process”, “immune system development”, “lipid oxidation” and “oxidoreductase activity” et al. (Figure S7c and Table S6). These functions indicated that even when not enriched in the DEGs, the antioxidative activity was still moderately induced or inhibited under FW acclimation.

## 3. Materials and Methods

### 3.1. Exposure of L. maculatus to Salinity Change and Alkalinity Stress

*L. maculatus* juveniles bred from seawater were acquired from Aquaculture Company (Yantai, Shandong Province, China) and acclimated in seawater (20 ± 1 °C, pH: 7.9 ± 0.2 and salinity 30 ppt) for three weeks. Carbonate-alkalinity solution was prepared by adding NaHCO_3_ (12.8 mmol/L) and Na_2_CO_3_ (2.6 mmol/L) to freshwater (pH: 7.4 ± 0.2) and aerating for 24 h before the experiment. The final pH for alkaline water was adjusted to 9.0 ± 0.2 by a pH meter and monitored every day. Because the alkalinity change needed to be started from freshwater, the seawater-originated *L. maculatus* were firstly fully acclimated from seawater to freshwater. Therefore, firstly, *L. maculatus* juveniles (158.23 ± 18.77 g) from seawater (SW, 30 ppt) were randomly transferred to freshwater (FW, 0 ppt) and brackish water (BW, 15 ppt) for 30 days [23], and then samples were collected for the SW, BW and FW groups, respectively. After FW acclimation, *L. maculatus* specimens (body weight: 140.32 ± 2.56 g) from FW were further transferred to alkaline water (AW, carbonate alkalinity: 18 ± 0.2 mmol/L) for 3 days, with the temperature and pH maintained at 20 ± 1 °C and pH 9.0 ± 0.2, respectively [26]. Due to the only moderate adaptation to alkaline water, *L. maculatus* specimens from the AW group were sampled at 0 h, 12 h, 24 h and 72 h. *L. maculatus* were anesthetized with MS-222 (Sigma-Aldrich, Darmstadt, Germany), and the gill tissues were rapidly sampled for both RNA extraction (−80 °C) and histological examination (Bouin’s fluid) after salinity change and alkalinity stress. Each group contained three tanks and three individuals from each tank were sampled as biological replicates.

### 3.2. Histological Examination of L. maculatus Gills

For histological observation, Bouin’s fluid was utilized for the preservation of gill samples and then dehydrated by a successive gradient of 50%, 70%, 90%, 95% and 100% ethanol. After transparentizing with xylene and alcohol mix, the gill samples were embedded in paraffin, sliced to a thickness of 5 µm, and then dewaxing and haematoxylin & eosin (H&E, Solarbio, Beijing, China) staining were performed in the standard manner [63]. The samples sealed with neutral gum were finally photographed using a Nikon Eclipse TiU microscope (Nikon, Tokyo, Japan) for observation.

### 3.3. Transcriptome Analysis of L. maculatus Gills under Salinity Change and Alkalinity Stress

To investigate the gene expression profiles of *L. maculatus* under stress, transcriptome data from gill tissues in response to salinity change (PRJNA515986) and alkalinity stress (PRJNA611641) were analyzed independently. Total RNA was extracted from gill samples and equal amounts of RNA from three individuals in the same tank were pooled as one sample. The RNA-seq of gill samples was carried out on the Illumina HiSeq X Ten platform with 150 bp paired-end reads. The RNA-seq raw data were quality checked and processed with the Hisat and StringTie pipeline (30 July 2022) [64]. Briefly, the clean reads were mapped to the *L. maculatus* genome (PRJNA408177) by Hisat with default parameters, and the Fragments Per Kilobase of exon per Million mapped reads (FPKM) of each gene were obtained by StringTie. Differential expression analysis (each test vs. control) was performed using the R package edgeR (10 August 2022) [65] to identify the differentially expressed genes (DEGs) with log_2_|Fold Change (FC)| ≥ 1.5 and *q value* < 0.05. TBtools [66] was employed to draw heatmaps with log_2_FC values (20 August 2022). Based on functional annotation of Gene Ontology (GO), GO terms at levels 3, 4, and 5 were enriched (*q value* < 0.05) in DEGs with EnrichPipeline (30 August 2022) [67].

### 3.4. Gene Co-Expression Network Construction and Functional Characterization

Weighted gene co-expression network analysis (WGCNA) [35] was employed to characterize the correlated gene expression patterns across the salinity- and alkalinity-challenged samples, from which the stress-responsive modules (SRMs) were identified by the WGCNA R library (15 September 2022) [68]. The gene dendrogram was used for module detection by the dynamic tree cut method (minimum module size = 50, cutting height = 0.99 and deepSplit = F). The hubness of a gene in a given module was determined by the intramodular connectivity (Kwithin), which measures a gene’s connection strength to other genes in the specified module. Each node (gene) was usually connected with several nodes through the edges with different weight values. The SRMs were identified based on the overrepresentation of genes using a hypergeometric test (*p* or *q value* < 0.05). GO terms were enriched at levels 3, 4, and 5 (*q value* < 0.05) for genes in each module by EnrichPipeline [67]. Cytoscape was employed for visualization of the co-expression network, which filtered the edges and focused on the nodes with the most tight edges [69].

## 4. Conclusions

**Figure 8:**
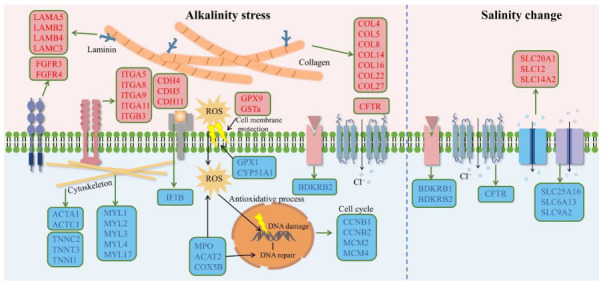
Schematic diagram revealing key genes and pathways involved in the response of *L. maculatus* gills to salinity change and alkalinity stress. The upregulated genes are mainly enriched in the extracellular matrix and cell adhesion molecules, which are shown in red font, while the downregulated genes were concentrated in the cytoskeleton, antioxidation, cell cycle and (ion)-transport, which are shown in blue font.

The diversified aquatic teleosts possess varied osmoregulatory responses reflecting in antioxidative, immune and cellular status. This study provided a systematic transcriptomic and gene co-expression network survey in Chinese sea bass (*L. maculatus*), which has good and moderate adaptability in saline and alkaline water, respectively. Diverse and specific gene expression regulation was observed in response to alkalinity stress but not in salinity change, which was mainly in the coordinately activated extracellular matrix and inhibited antioxidative and immune functions (Figure 8). These findings are useful for understanding the functions of gene networks and adaptive evolution in teleost species. Further investigation on the function of SRM networks will provide a more detailed explanation of their adaptation mechanisms against different environmental challenges and improve the feasibility of the cultivation of *L. maculatus* in saline-alkaline water.

## Data Availability

The *L. maculatus* transcriptome datasets used in this study can be found in the NCBI Sequence Read Archive (SRA) BioProject PRJNA611641 and PRJNA515986.

## Supplementary Materials

The following supporting information can be downloaded at: https://www.mdpi.com/article/10.3390/ijms24065877/s1.
