# Supplementary material for: Transcriptomic Modulation Reveals the Specific Cellular Response in Chinese Sea Bass (Lateolabrax maculatus) Gills under Salinity Change and Alkalinity Stress
Source: Int J Mol Sci. 2023 Mar 20;24(6):5877. doi: 10.3390/ijms24065877 (PMC10056482; doi:10.3390/ijms24065877)
Supplement: Supplementary file 1 [file ijms-24-05877-s001.zip › Supplementary Figures and Tables Legends.pdf]

Figure S1: Differential gene expression and functional GO enrichment of DEGs in *L. maculatus* gills under salinity change. (**a**) Heat map for expressed DEGs; (**b**) Number of DEGs between groups; (**c**) DEGs shown as the Venn diagram between the salinity groups; (**d**) Functional GO enrichment of the salinity DEGs at level 3.

Figure S2: Differential gene expression and functional GO enrichment of DEGs in *L. maculatus* gills under alkalinity stress. (**a**) Heat map for expressed DEGs; (**b**) Number of DEGs between groups; (**c**) DEGs shown as the Venn diagram between the alkalinity groups; (**d**) Functional GO enrichment of the alkalinity DEGs at level 3.

Figure S3: Expression profiles of the overlapped DEGs in response to salinity change and alkalinity stress.

Figure S4: Modulation from the WGCNA.

Figure S5: Expression profiles of some functional DEGs in response to salinity change and alkalinity stress. (**a**) transport related DEGs; (**b**) ECM related DEGs; (**c**) filament related DEGs.

Figure S6: The GO enrichment and gene network of alkaline specific SRMs in *L. maculatus* gills, as the (**a**) blue, (**b**) royalblue and (**c**) lightyellow modules with top 300 genes ranking with their connectivity. The circle size represents the intramodular connectivity (Kwithin), and the circle frame color represents the annotated GO terms; ** indicate $P < 0.001$.

Figure S7: The GO enrichment and gene network of saline specific SRMs in *L. maculatus* gills, as the (**a**) cyan, (**b**) black and (**c**) darkgreen modules with top 300 genes ranking with their connectivity. The circle size represents the intramodular connectivity (Kwithin), and the circle frame color represents the annotated GO terms; * indicate $P < 0.05$ and ** indicate $P < 0.001$.

Table S1: GO enrichment of the DEGs from *L. maculatus* gills under salinity change and alkalinity stress, respectively.

Table S2: GO enrichment of the DEGs from the combined salinity and alkalinity stresses of *L. maculatus* gills.

Table S3: General information for the modules of WGCNA.

Table S4: Identification of SRMs.

Table S5: Summarized information of the conventional *L. maculatus* antioxidative genes.

Table S6: GO enrichment of each SRM.
