# Supplementary material for: Transcriptomic Modulation Reveals the Specific Cellular Response in Chinese Sea Bass (Lateolabrax maculatus) Gills under Salinity Change and Alkalinity Stress
Source: Int J Mol Sci. 2023 Mar 20;24(6):5877. doi: 10.3390/ijms24065877 (PMC10056482; doi:10.3390/ijms24065877)
Supplement: Supplementary file 1 [file ijms-24-05877-s001.zip › Table S5.Oxi gene info.new.pdf]

	Family	genes	No. of Exons	Gene ID	Scaffold ID	Start	End	Gene size (bp)	Strand
**sod**		sod1	5	GLEAN_10001859	scaffold695	133545	137402	3857	-
		sod2	5	GLEAN_10012279	scaffold102	1457470	1459545	2075	+
		sod3b	1	GLEAN_10015360	scaffold199	2137637	2138269	632	-
**gpx**	gpx1	gpx1a1	2	GLEAN_10018729	scaffold96	4129631	4130954	1323	-
		gpx1a2	1	GLEAN_10020004	C5878552	1	192	191	-
		gpx1a3	1	GLEAN_10020005	C6483015	1	123	122	-
	gpx2	gpx2a	1	GLEAN_10017570	scaffold163	785498	785878	380	-
		gpx2b	1	GLEAN_10020409	scaffold163	786401	786511	110	-
	gpx3	gpx3	4	GLEAN_10018137	scaffold217	3788394	3820319	31925	+
	gpx4	gpx4a1	6	GLEAN_10020465	scaffold50	71373	73307	1934	+
		gpx4a2	4	GLEAN_10020464	scaffold50	71636	73307	1671	+
	gpx7	gpx7	7	GLEAN_10014809	scaffold17	32089	68691	36602	+
	gpx9	gpx9	5	GLEAN_10009555	scaffold192	942268	943799	1531	-
**cat**	cat	cat	14	GLEAN_10012199	scaffold147	1237805	1251044	13239	+
**gst**	gst	gsta	6	GLEAN_10017743	scaffold43	553487	556683	3196	-
		gstk1	4	GLEAN_10000407	C7150537	288	1167	879	-
		gstm4	8	GLEAN_10017280	scaffold60	699378	702295	2917	+
		gstr	13	GLEAN_10013168	scaffold584	485940	502753	16813	+
		gstr2	6	GLEAN_10013167	scaffold584	480702	483074	2372	+
		gstt1	4	GLEAN_10015211	scaffold216	1713405	1716927	3522	+
		gstt2	5	GLEAN_10002723	scaffold162	6228	8525	2297	+
	mgst	mgst1	3	GLEAN_10003971	scaffold442	150081	151908	1827	+
		mgst2	7	GLEAN_10002172	scaffold1055	58981	65147	6166	+
		mgst3	7	GLEAN_10007782	scaffold432	251768	255620	3852	-
		mgst3b	4	GLEAN_10017674	scaffold163	3539044	3540462	1418	+
**cyp**	cyp1	cyp1a	9	GLEAN_10010754	scaffold205	88714	95044	6330	-
		cyp1b1*	2	GLEAN_10016652	scaffold172	2119967	2122220	2253	-
		cyp1c1	1	GLEAN_10017593	scaffold163	1318492	1320069	1577	+
		cyp1c2	1	GLEAN_10017594	scaffold163	1322543	1324117	1574	+
		cyp1d1	8	GLEAN_10019218	scaffold86	7538085	7542792	4707	-
	cyp2	cyp2f2	20	GLEAN_10005479	scaffold21	478751	499000	20249	-
		cyp2j2	9	GLEAN_10015623	scaffold38	200952	204333	3381	+
		cyp2k1	16	GLEAN_10004424	scaffold639	41029	58701	17672	+
		cyp2n13	18	GLEAN_10004065	scaffold132	379617	392417	12800	-
		cyp2r1	5	GLEAN_10009810	scaffold68	712491	715624	3133	+
		cyp2y3	16	GLEAN_10015567	scaffold23	1525644	1538767	13123	+
	cyp3	cyp3a30	13	GLEAN_10016462	scaffold229	385065	390525	5460	+
		cyp3a65	12	GLEAN_10013957	scaffold19	334444	339309	4865	-
	cyp4	cyp4f3	22	GLEAN_10004161	scaffold455	12660	22142	9482	+
		cyp4v8	11	GLEAN_10014003	scaffold19	1396427	1403682	7255	+
	cyp19	cyp19a1	9	GLEAN_10006463	scaffold24	71824	75004	3180	+
	cyp51	cyp51a1	10	GLEAN_10003044	scaffold202	60954	64485	3531	-
