# Supplementary figures and images for: Transcriptomic Modulation Reveals the Specific Cellular Response in Chinese Sea Bass (Lateolabrax maculatus) Gills under Salinity Change and Alkalinity Stress
Source: Int J Mol Sci. 2023 Mar 20;24(6):5877. doi: 10.3390/ijms24065877 (PMC10056482; doi:10.3390/ijms24065877)

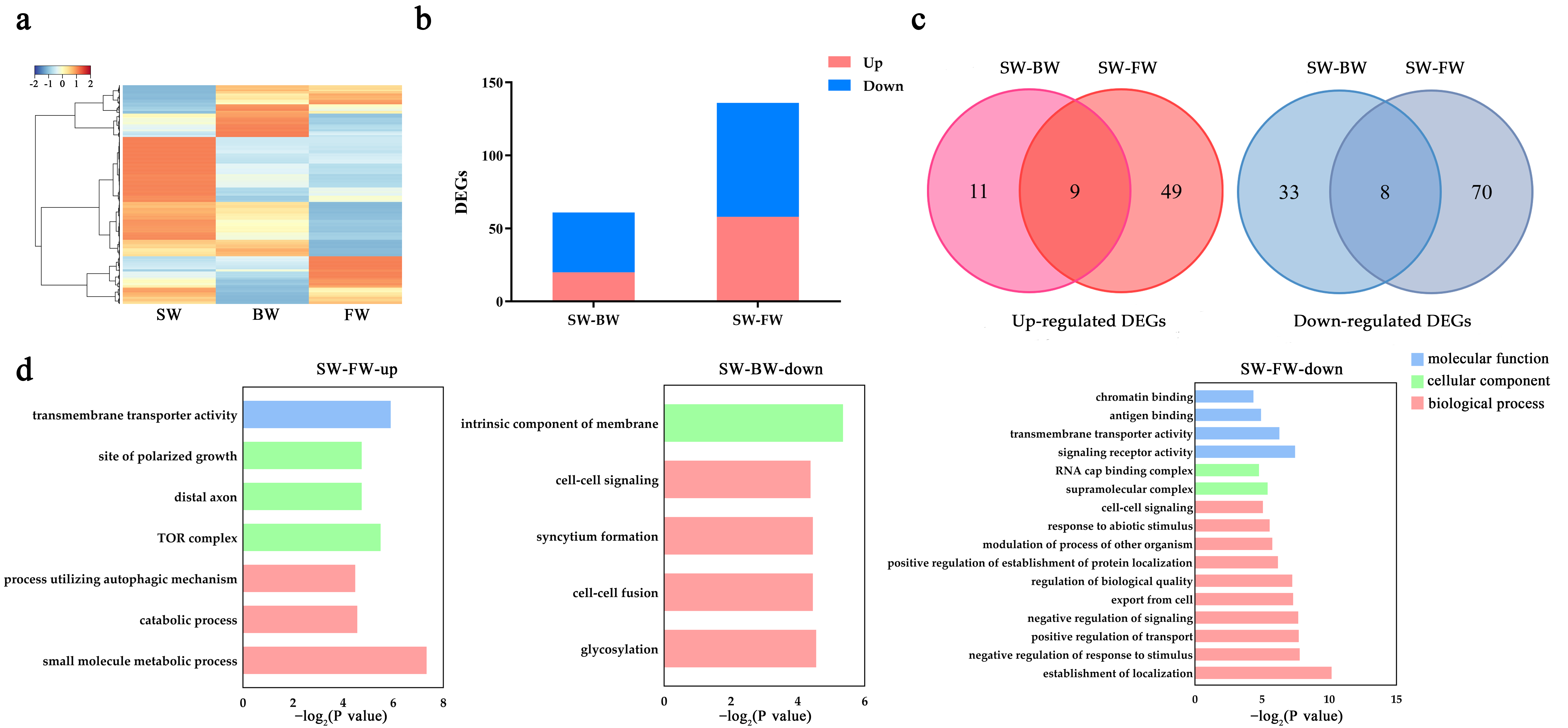

Supplement: Supplementary file 1 [file ijms-24-05877-s001.zip › Figure S1.Salinity-DEG-1.5new.tif]

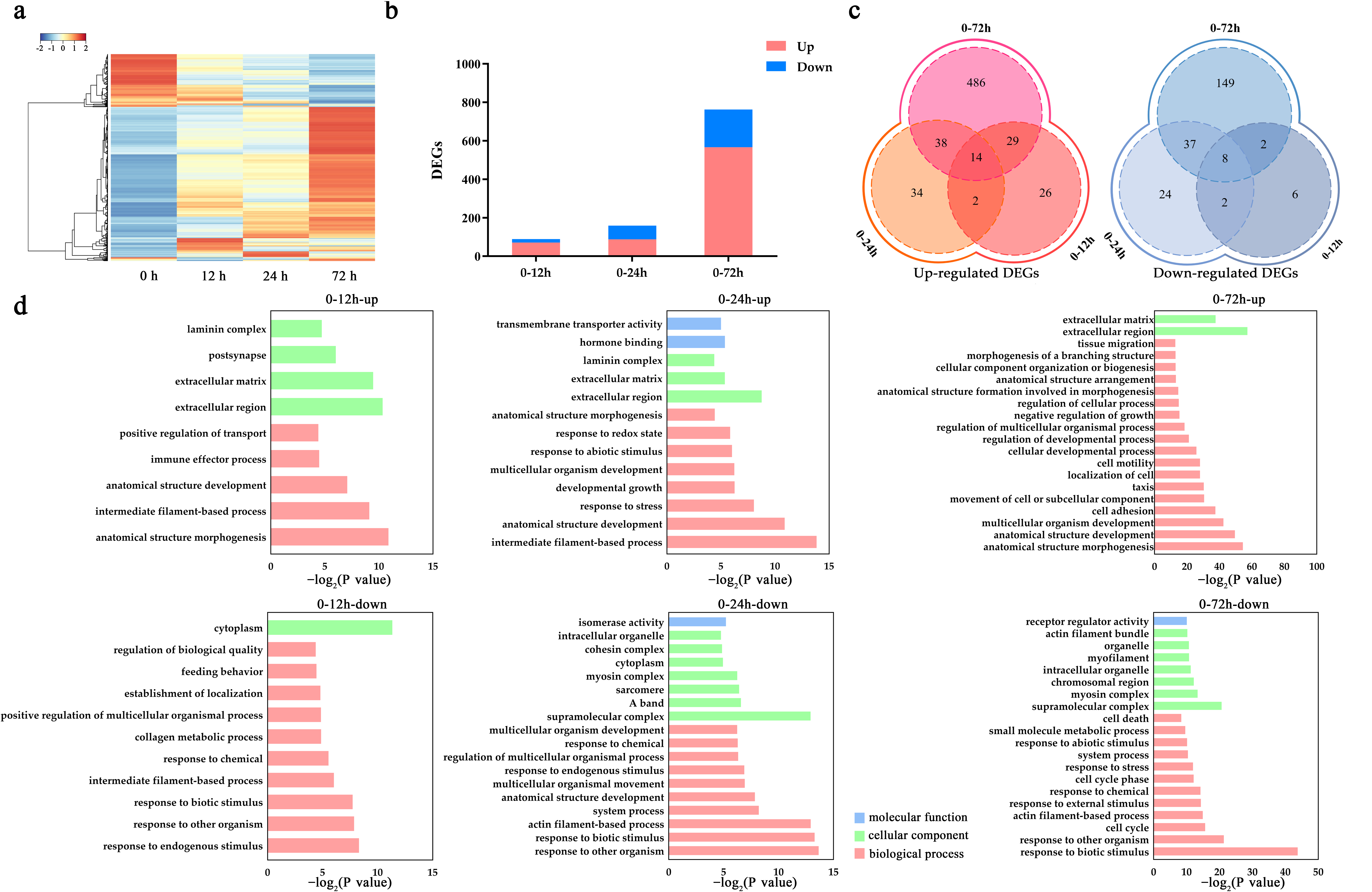

Supplement: Supplementary file 1 [file ijms-24-05877-s001.zip › Figure S2.Alkalinity-DEG-1.5new.tif]

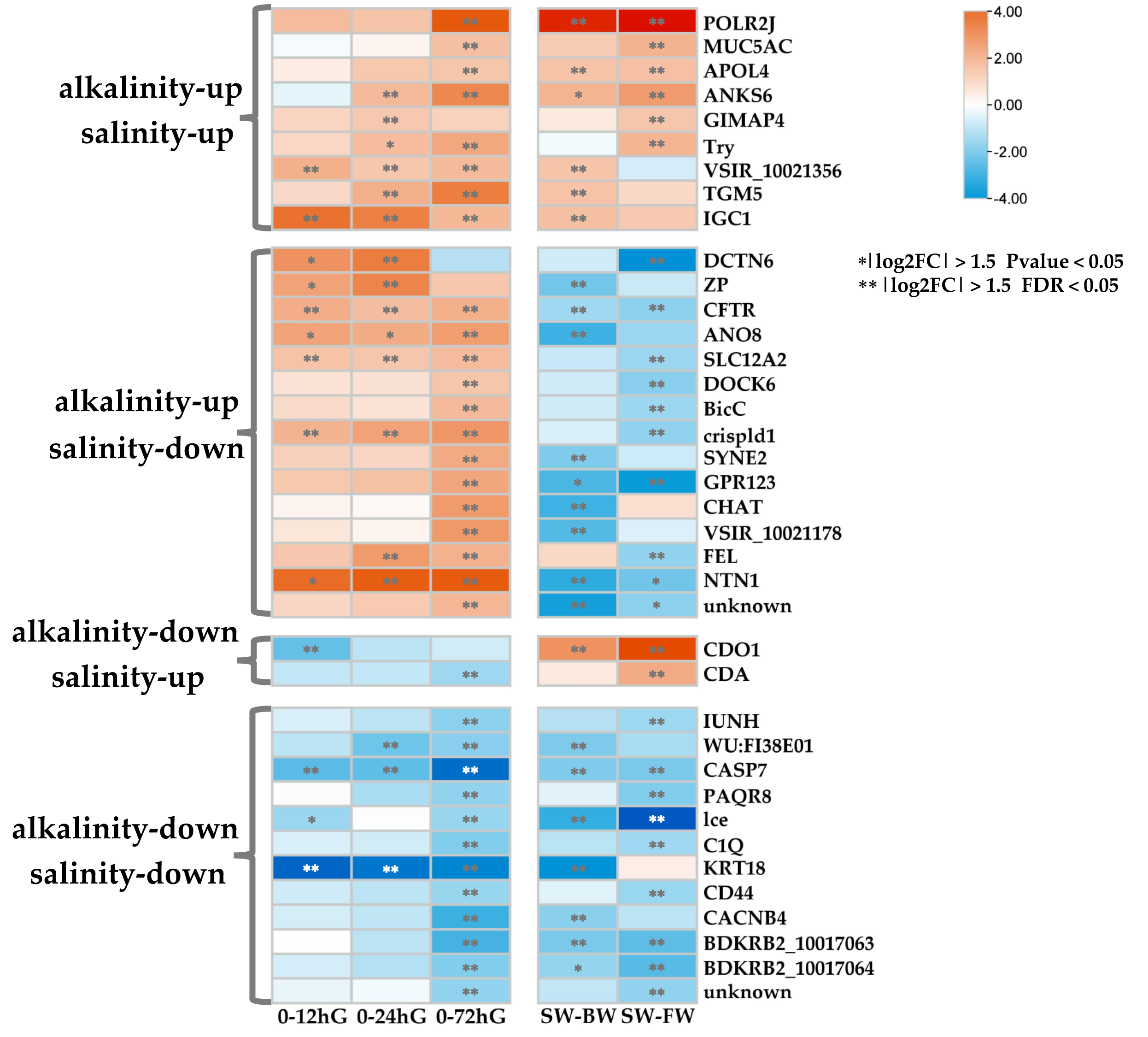

Supplement: Supplementary file 1 [file ijms-24-05877-s001.zip › Figure S3.Overlapped DEG HeatMap.tif]

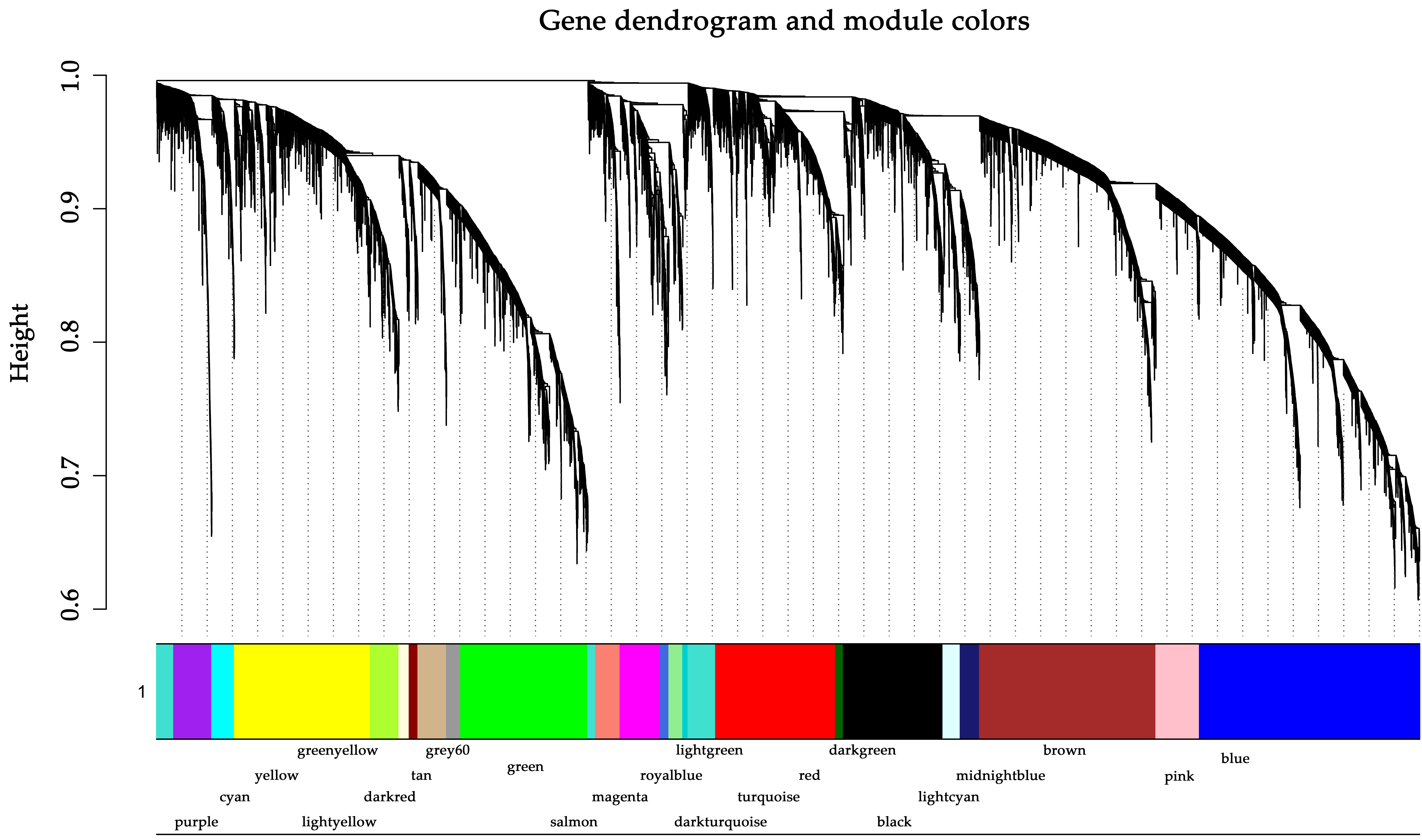

Supplement: Supplementary file 1 [file ijms-24-05877-s001.zip › Figure S4.WGCNA.tif]

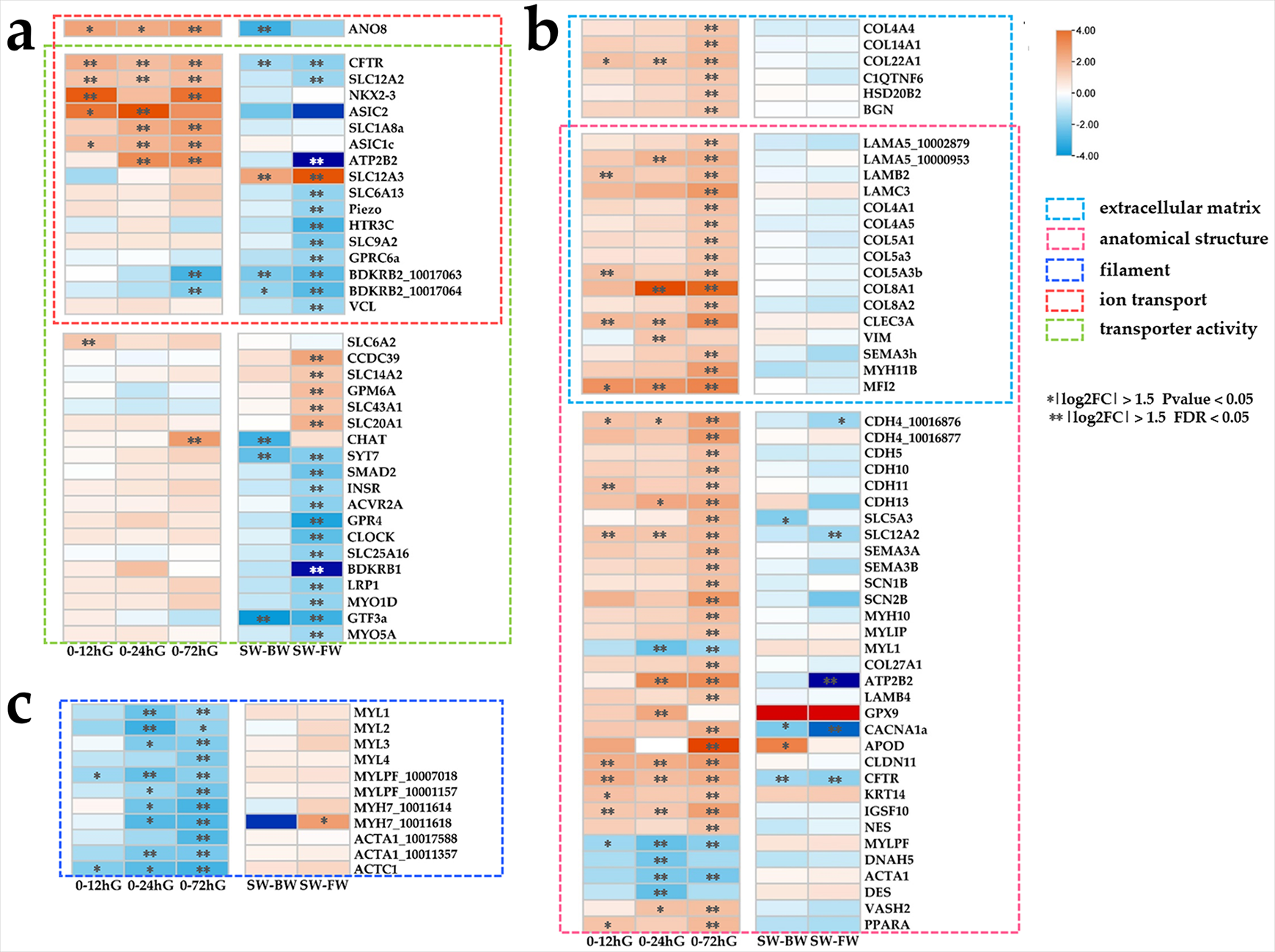

Supplement: Supplementary file 1 [file ijms-24-05877-s001.zip › Figure S5.GO DEG HeatMap3(小).tif]

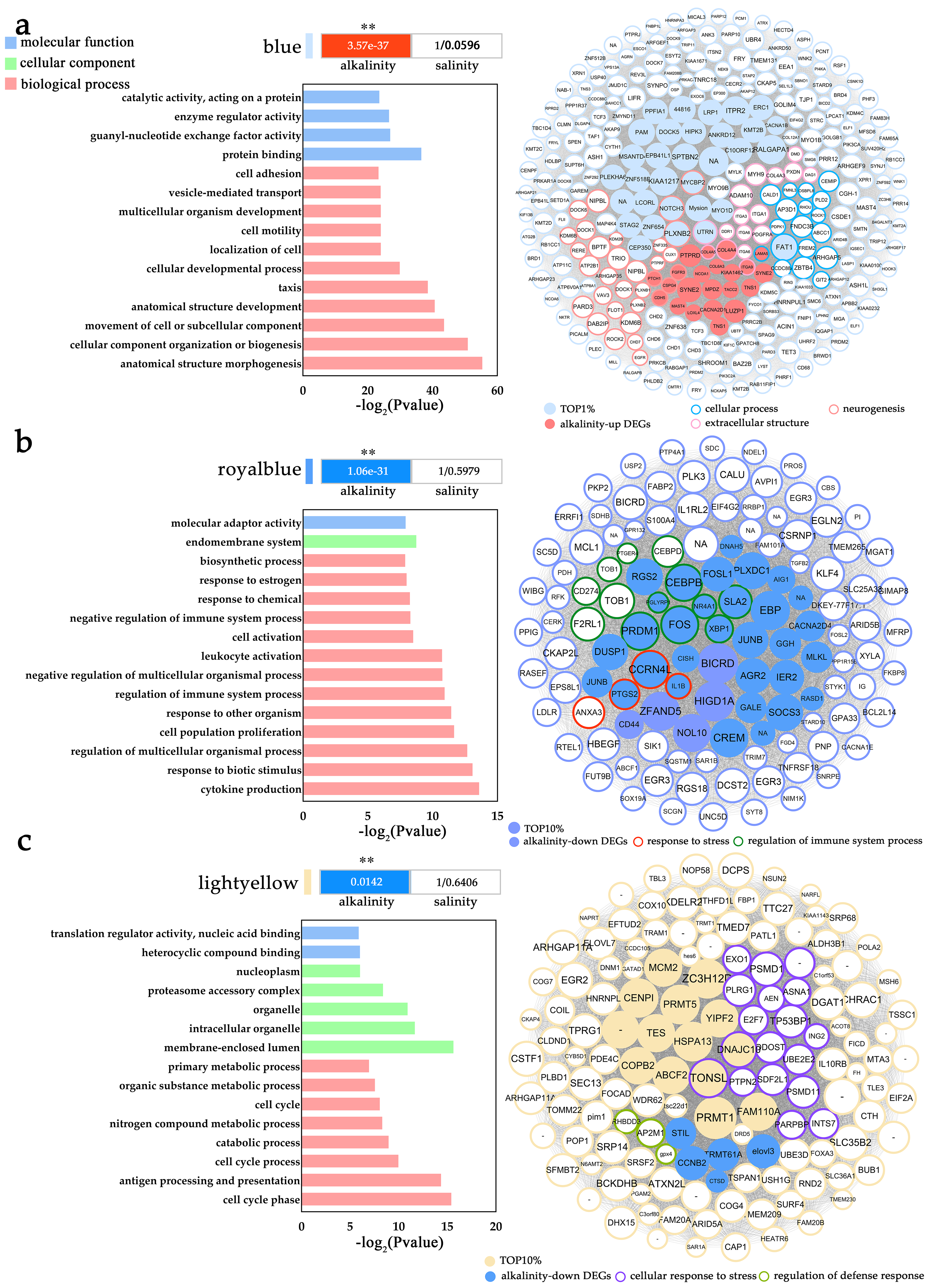

Supplement: Supplementary file 1 [file ijms-24-05877-s001.zip › Figure S6.Alka SRMs.new.tif]

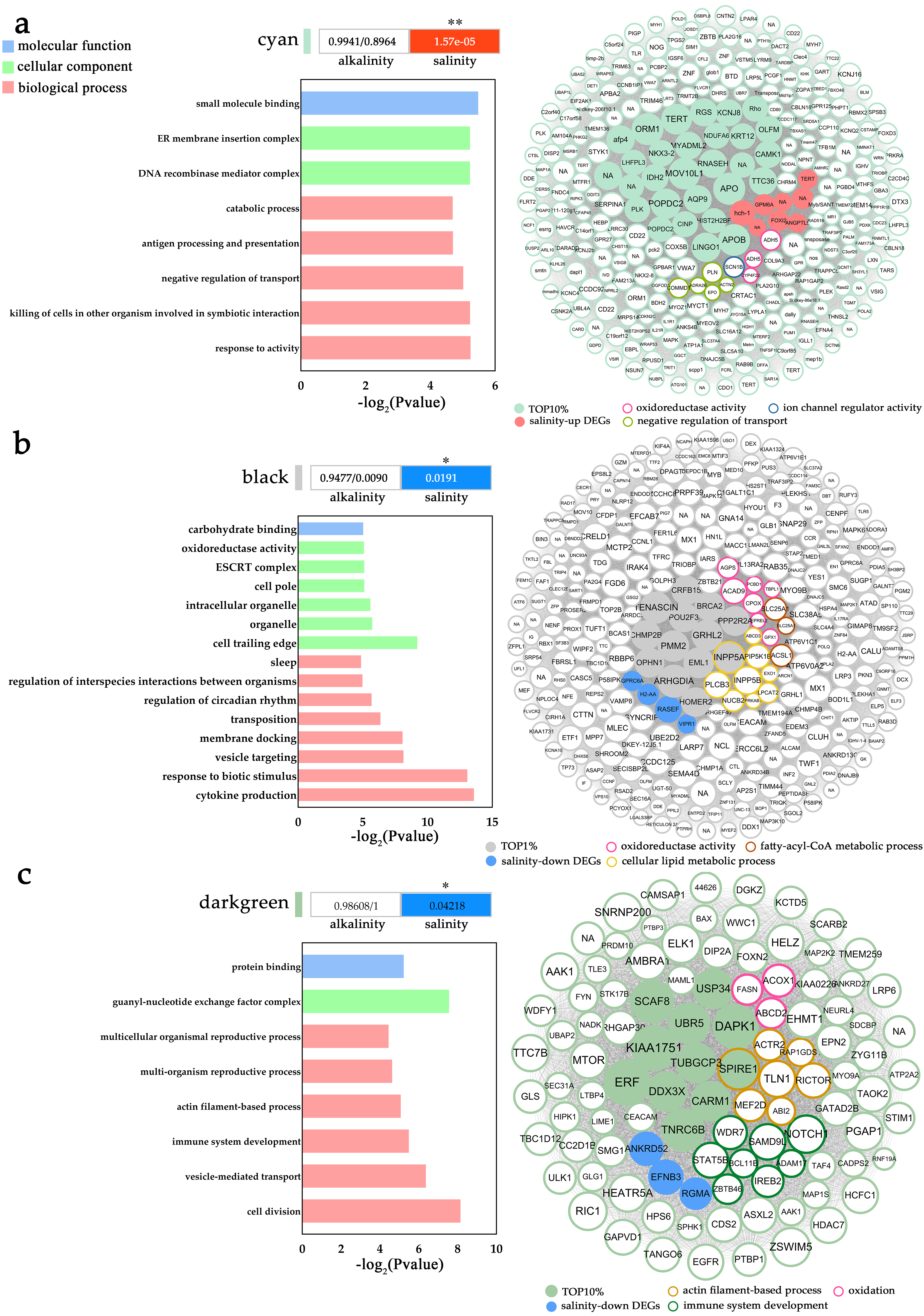

Supplement: Supplementary file 1 [file ijms-24-05877-s001.zip › Figure S7.Sali SRMs.new.tif]
